# Scale-Aware Tracking Method with Appearance Feature Filtering and Inter-Frame Continuity

**DOI:** 10.3390/s23177516

**Published:** 2023-08-30

**Authors:** Haiyu He, Zhen Chen, Zhen Li, Xiangdong Liu, Haikuo Liu

**Affiliations:** 1 School of Automation, Beijing Institute of Technology, Beijing 100010, China; 3220195103@bit.edu.cn (H.H.); chenzhen76@bit.edu.cn (Z.C.); zhenli@bit.edu.cn (Z.L.); xdliu@bit.edu.cn (X.L.); 2 School of Mechatronical Engineering, Beijing Institute of Technology, Beijing 100010, China

**Keywords:** discriminative correlation filter, scale estimation, color name, salient feature, visual tracking

## Abstract

Visual object tracking is a fundamental task in computer vision that requires estimating the position and scale of a target object in a video sequence. However, scale variation is a difficult challenge that affects the performance and robustness of many trackers, especially those based on the discriminative correlation filter (DCF). Existing scale estimation methods based on multi-scale features are computationally expensive and degrade the real-time performance of the DCF-based tracker, especially in scenarios with restricted computing power. In this paper, we propose a practical and efficient solution that can handle scale changes without using multi-scale features and can be combined with any DCF-based tracker as a plug-in module. We use color name (CN) features and a salient feature to reduce the target appearance model’s dimensionality. We then estimate the target scale based on a Gaussian distribution model and introduce global and local scale consistency assumptions to restore the target’s scale. We fuse the tracking results with the DCF-based tracker to obtain the new position and scale of the target. We evaluate our method on the benchmark dataset Temple Color 128 and compare it with some popular trackers. Our method achieves competitive accuracy and robustness while significantly reducing the computational cost.

## 1. Introduction

Visual object tracking is a fundamental research topic in computer vision and pattern recognition that has many applications, such as robot vision [1,2], video surveillance [3,4,5], medical–industrial integration [6,7,8], etc. The main task of visual tracking is to estimate the target’s position and scale according to the given target specified by a bounding box in the first frame. Trackers need to achieve both high accuracy and efficiency to be feasible for many applications [9]. However, visual object tracking faces many challenges that affect its performance and robustness. Scale variation, which occurs when the target changes its size or distance from the camera, is one of the most difficult challenges that affects the performance of trackers [10,11].

DCFs are effective trackers that achieve high accuracy and efficiency by transforming the matching process from the spatial domain to the frequency domain [12]. However, DCF cannot estimate the scale change [13] by itself. Since DCF-based methods are based on templates used to locate objects, long image processing time will lead to tracking failure due to boundary effects [14]. Existing scale estimation methods based on multi-scale features are computationally intensive and degrade the real-time performance of DCF-based trackers, especially in scenarios with limited computational power [15].

In this paper, to address the above issues, we propose a computationally efficient and scale-adaptive tracking method based on DCF and probability estimation. The main contributions of this paper can be summarized as follows:

(1) We propose a practical scale-adaptive tracking method based on probability estimation (PSACF), which can handle scale changes without using multi-scale features and can be combined with other tracking methods as a plug-in. We also adopt a fusion tracking strategy to fuse the results with the position localization results.

(2) We propose a local adaptive salient feature and scale restoration method, which selects the local salient features of the target in the current image and reduces the interference caused by background clutter. Based on global and local scale consistency assumptions, we perform global scale recovery based on the Gaussian model and inter-frame continuity of salient features, which improves the accuracy and robustness of scale estimation.

The rest of this paper is organized as follows. Section 2 introduces related works, Section 3 revisits the related formulation, and Section 4 presents the detailed design. Experiments are conducted to verify the proposed algorithm in Section 4. Finally, Section 6 gives the main conclusions.

## 2. Related Works

Visual tracking has been studied extensively over the past decades. In this section, we discuss the methods closely related to this work in terms of DCF-based trackers and scale estimation methods.

### 2.1. DCF-Based Trackers

DCFs are effective trackers that achieve high accuracy and efficiency by transforming the matching process from the spatial domain to the frequency domain. During the past decades, a variety of tracking algorithms based on DCF have been proposed [10,13,16,17,18,19]. The minimum output sum square error (MOSSE) tracker proposed by Bolme et al. [20] showed the strong potential of DCFs, whose processing speed reached 669 FPS (frames per second). However, MOSSE only utilized gray features, which limited its performance in complex scenarios. To address this limitation, Henriques et al. [13] exploited the cyclic structure with a kernel (CSK) based on MOSSE. By doing so, they extended the gray features to multichannel HOG features and proposed the kernel correlation filter (KCF), which improved the tracking performance while maintaining a high processing speed of 172 FPS. However, both MOSSE and KCF suffered from boundary effects, which caused unwanted background information to be included in the target model. To address this problem, Danelljan et al. [16] proposed spatially regularized DCFs (SRDCF), which introduced a spatial regularization term to penalize filter coefficients corresponding to background regions. SRDCF achieved superior performance over KCF, but at the cost of reduced speed.

One of the problems of DCF-based trackers is that they cannot handle scale variation by themselves. In order to estimate the target scale, they need to use additional methods or components, which we review in the next subsection.

### 2.2. Scale Estimation Methods

Scale variation is one of the most challenging problems in visual tracking. A tracker that cannot handle scale variation may lose the target or drift to the background when the target changes its size or distance from the camera.

One common way to estimate the scale is to exploit multi-scale features. For example, discriminative scale space tracker (DSST) [10] and scale adaptive with multiple features tracker (SAMF) [11] trained a separate scale classifier by constructing a multi-scale image pyramid for the detection of target scale variation. They first detected the target position by DCF, and then extracted multi-scale features in the predicted position regions to estimate the scale using another DCF. However, these methods were computationally expensive and slowed down the tracker, especially in scenarios with restricted computing power.

Another way to estimate the scale is to traverse the scale space to match the target or train a scale classifier. Ma et al. [21] proposed a scale searching scheme that focused on the response map and incorporated the average peak-to-correlation energy criterion into a multi-resolution translation filter framework to handle scale variation. Lu et al. [22] applied an additional correlation filter over scale-aware region proposals for scale estimation. They built a region proposal algorithm based on EdgeBox by adopting a support vector machine (SVM) classifier to obtain a set of proposals for scale and position detection. Zhang et al. [18] proposed a multitask correlation particle filter for robust visual tracking. They adopted a particle sampling strategy to address the scale variation issue. Vojir et al. [23] proposed scale-adaptive mean-shift (ASMS), in which they adopted a scale estimation method and achieved a relative balance between the tracking accuracy and computational efficiency. There are also some methods that use deep neural networks to tackle the scale variation problem [24,25,26,27], but deep learning-based methods heavily rely on GPUs and are difficult to apply in scenarios with limited computing resources.

However, these methods still relied on some form of multi-scale feature or multi-scale traversing strategy, which increased the computational cost and complexity of the tracker. Moreover, they did not consider the inter-frame continuity or consistency of appearance between frames, which could significantly reduce the computation cost. We exploit the distribution of the salient feature of the appearance and employ the probability estimation method in this paper to tackle the scale variation problem. Our method achieves competitive accuracy and robustness while significantly improving the processing speed over existing methods.

## 3. Discriminative Correlation Filter

In this paper, the KCF [13] is adopted for its efficiency. In this section, we briefly review the KCF tracking method with the fundamental formulas. The proposed scale estimation method is detailed in the next section.

The core idea of the DCF algorithm is to train a classifier via the ridge regression method by minimizing the squared error over the samples $x_{i}$ and their regression targets $y_{i}$,

(1) $$\min_{w}\sum_{i}{\left(f\left(x_{i}\right)-y_{i}\right)}^{2}+\lambda {∥w∥}^{2}.$$

The minimizer has a closed form, which is given by

(2) $$w={\left(X^{H}X+\lambda I\right)}^{-1}X^{T}y,$$

where *X* is the data matrix, and $X^{H}$ is the Hermitian transpose. For nonlinear regression, the kernel trick is applied to the model

(3) $$w=\sum_{i}\alpha_{i}\varphi \left(x_{i}\right),$$

where α is the vector of coefficients $\alpha_{i}$, and $\varphi \left(x_{i}\right)$ is the arbitrary nonlinear mapping of $x_{i}$. The filter can be accelerated via discrete Fourier transform (DFT) as

(4) $$\hat{\alpha }=\frac{\hat{y}}{{\hat{k}}^{xx}+\lambda },$$

where ${\hat{k}}^{xx}$ is the cross-correlation in the high-dimensional space φ(x). In this paper, the Gaussian kernel is adopted as

(5) $$k^{{\mathrm{xx}}^{\prime }}=\exp\left(-\frac{1}{\delta^{2}}\left({∥x∥}^{2}+{∥x^{\prime }∥}^{2}-2F^{-1}\left(\hat{x}⊙{\hat{x}}^{\prime *}\right)\right)\right),$$

where $F^{-1}$ denotes the inverse FFT transform. The response in the Fourier domain can be calculated as

(6) $$\hat{f}\left(z\right)={\left({\hat{k}}^{xz}\right)}^{*}⊙\hat{\alpha }.$$

When transformed back into the spatial domain, the response map denotes the location of the target being tracked.

## 4. Proposed Algorithm

**Figure 1:**
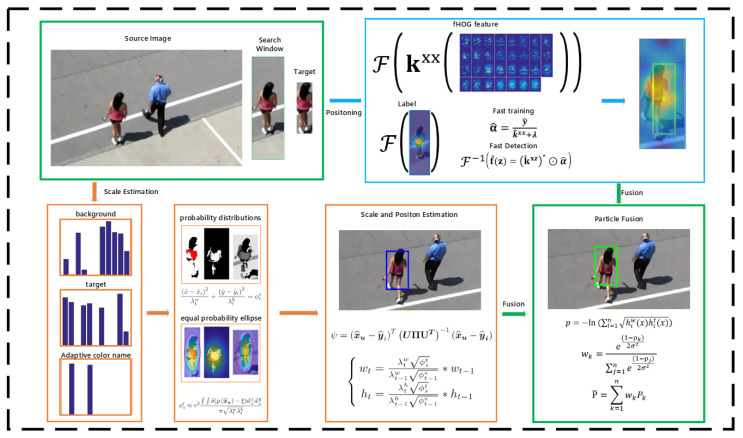
Overview of the proposed method.

In this section, an introduction to our tracker is briefly given. Additionally, we present the overall process of our salient feature filtering and scale estimation scheme in detail. Finally, the mechanism of result fusion and the strategy of template updating are discussed. An overview of the proposed method is given in Figure 1, and the pseudo-algorithm is shown in Algorithm 1.
**Algorithm 1** PSACF: Probability Estimation and Scale-Adaptive Correlation Filter Tracking**Input:** The first frame $I_{1}$ and the target region $R_{1}$**Output:** The target region $R_{t}$ in each frame $I_{t}$
  1:Initialize the target appearance model $H^{t}$ and the background appearance model $H^{b}$ based on the CN feature  2:Initialize the target position $P_{1}$ and scale $S_{1}$ based on the target region $R_{1}$  3:**for** *t* = 2,3,… **do**  4:    Extract the search window $W_{t}$ around the target position $P_{t-1}$ in the frame $I_{t}$  5:    Calculate the salient feature $H^{t}$ of the target based on Equation (10)  6:    Estimate the probability $p(\hat{x},\hat{y})$ of each candidate pixel in the search window based on Equation (15)  7:    Weight the probability $\hat{p}\left(\hat{x},\hat{y}\right)$ of each candidate pixel based on Equation (17)  8:    Estimate the scale $S_{t}$ of every salient feature based on Equations (24) and (26)  9:    Update the target appearance model $H^{t}$ based on Equation (32)  10:    Obtain two position candidates $P_{tc}$ and $P_{tk}$ from the proposed algorithm and the KCF algorithm, respectively  11:    Calculate the weights $w_{tc}$ and $w_{tk}$ of each position candidate based on Equation (31)  12:    Fuse the position candidates to obtain the final position estimate $P_{t}=w_{tc}P_{tc}+w_{tk}P_{tk}$  13:    Output the target region $R_{t}=\left(P_{t},S_{t}\right)$ in the frame $I_{t}$  14:**end for**

### 4.1. Fast Adaptive Salient Feature Filtering Algorithm

In this paper, the CN feature is adopted as the appearance feature. The CN features are based on a set of 11 basic color names: black, blue, brown, gray, green, orange, pink, purple, red, white, and yellow. Each pixel in the image is assigned a probability distribution over these 11 color names based on a probabilistic model trained on a large dataset of natural images. The model used is provided by [28].

The CN histogram includes 11-dimensional features, most of which are invalid or are interference items from the background. During tracking, these invalid features in the background will interfere with the target. To reduce the influence of these features, the weight of these features in the target histogram needs to be suppressed or deleted. In the process, three histograms are involved, i.e., the feature histogram of the target, the feature histogram of the background, and the salient feature histogram of the target.

Firstly, the histogram of the target $H^{t}$ and the histogram of the whole search window $H^{a}$ are modeled. Additionally, the background histogram $H^{b}$ is defined. It is obvious that the histogram of the whole search window is divided into the arithmetic sum of the target histogram and the background histogram:

(7) $$\begin{array}{cc} H^{t} & =\left[\begin{array}{cccc} h_{1}^{t}\left(x\right), & h_{2}^{t}\left(x\right), & \cdots , & h_{1}^{t}\left(x\right) \end{array}\right], \end{array}$$

(8) $$\begin{array}{cc} H^{a} & =\left[\begin{array}{cccc} h_{1}^{a}\left(x\right), & h_{2}^{a}\left(x\right), & \cdots , & h_{n}^{a}\left(x\right) \end{array}\right], \end{array}$$

(9) $$\begin{array}{cc} H^{b} & =H^{a}-H^{t}. \end{array}$$

The back-projection is then applied to the search window in order to obtain the target candidate probability distribution. Accordingly, the target histogram is rebuilt by

(10) $${\hat{h}}_{i}^{t}\left(x\right)=C\sum_{i=1}^{n}\frac{\ln\left(\frac{h_{i}^{a}-h_{i}^{t}+1}{h_{i}^{t}+1}\right)}{{\left(x_{i}-x_{c}\right)}^{2}}h_{i}^{t}\left(x_{i}\right)M\left(x_{i}\right),$$

where $h_{i}^{t}\left(x\right)$ denotes the *i*-th bin of the salient feature histogram of the target, $x_{i}$ denotes the pixel position, and $x_{c}$ is the center of the search window. *n* is the number of pixels in the search window, $h_{i}^{a}\left(x\right)$ is the *i*-th bin of the search window, *C* is a normalization constant, and M(x) maps the value of the pixel at location *x* to the corresponding bin in the CN space. The above process can be regarded as a weighting process. ${\left(x_{i}-x_{c}\right)}^{2}$ denotes the distance between the pixel location and the center of the search window, and we utilize this distance to lower the importance of edge pixels. $h_{i}^{t}\left(x_{i}\right)$ represents the probability that the pixel at $x_{i}$ belongs to the target.

Therefore, the salient feature histogram of the target can be further described as:

(11) $${\hat{H}}^{t}=\left[\begin{array}{cccc} {\hat{h}}_{1}^{t}\left(x\right), & {\hat{h}}_{2}^{t}\left(x\right), & \cdots , & {\hat{h}}_{n}^{t}\left(x\right) \end{array}\right].$$

In some application scenarios, there is no solution to (10). As a result, we choose the last two bins as the salient features.

### 4.2. Scale Estimation

The candidate pixel at ${\hat{x}}_{u}={\left(\hat{x},\hat{y}\right)}^{T}$ is defined as $I(\hat{x},\hat{y})$. It is worth mentioning that there still exists a partial background in the target bounding box, which causes interference in scale estimation. Therefore, the probability $p\left({\hat{x}}_{u}\right)$ of whether $I(\hat{x},\hat{y})$ belongs to the target needs to be estimated:

(12) $${\hat{q}}_{u}=C\sum_{i=1}^{n}K\left({∥x_{i}∥}^{2}\right)\delta \left(h\left(x_{i}\right)-u\right),u=1,\cdots ,m,$$

where δ is the Kronecker delta, and *i* denotes the pixel location. $h\left(x_{i}\right)$ is the corresponding bin in the feature space of the *i*-th pixel, $K\left({∥x_{i}∥}^{2}\right)$ is the RBF kernel function [23], and *C* is a normalization constant.

According to Formula (12), the probability can be estimated as follows based on the Bayesian formula

(13) $$\begin{array}{c} p\left(\hat{x},\hat{y}\right)=p\left(I\in H^{t}|I\notin H^{b}\right)=\frac{p\left(I\in H^{t}\right)*p\left(I\notin H^{b}|I\in H^{t}\right)}{p\left(I\notin H^{b}\right)}, \end{array}$$

where $I\in H^{t}$ denotes the event that pixel *I* belongs to the target, and $I\notin H^{b}$ denotes the event that *I* does not belong to the background in the search window.

When screening for salient features, the background features in the search window have been suppressed; therefore,

(14) $$\begin{array}{c} p\left(I\notin H^{b}|I\in H^{t}\right)=\frac{p\left(I\notin H^{b},I\in H^{t}\right)}{p\left(I\in H^{t}\right)}=\frac{p\left(I\in {\hat{H}}^{t}\right)}{p\left(I\in H^{t}\right)}, \end{array}$$

and Formula (13) can be described as

(15) $$\begin{array}{c} p\left(\hat{x},\hat{y}\right)=\frac{p\left(I\in H^{t}\right)*p\left(I\notin H^{b}|I\in H^{t}\right)}{p\left(I\notin H^{b}\right)}=\frac{p\left(I\in {\hat{H}}^{t}\right)}{1-p\left(I\in H^{b}\right)}. \end{array}$$

Meanwhile, the farther the pixel is from the center, the lower the probability that it belongs to the target; thus, we define the distribution of candidate pixels in the search window as a Gaussian model:

(16) $$N\left({\hat{x}}_{u},{\hat{y}}_{i},\Psi \right)=\frac{1}{\Phi }\exp\left(-\frac{1}{2}{\left({\hat{x}}_{u}-{\hat{y}}_{i}\right)}^{T}\Psi^{-1}\left({\hat{x}}_{u}-{\hat{y}}_{i}\right)\right),$$

where ${\hat{y}}_{i}={\left({\hat{x}}_{i},{\hat{y}}_{i}\right)}^{T}$ is the target position in the *i*-th frame, Ψ represents the shape of the target, and $\Phi ={\left(2\pi \right)}^{\frac{1}{2}}{∥\Psi ∥}^{\frac{1}{2}}$. It is obvious that candidate pixel distribution is jointly determined by variables ${\hat{x}}_{u},{\hat{y}}_{i}$, and Ψ. Therefore, the probability $p\left({\hat{x}}_{u}\right)$ should be weighted by distribution model $N\left({\hat{x}}_{u},{\hat{y}}_{i},\Psi \right)$,

(17) $$\hat{p}\left({\hat{x}}_{u}\right)=N\left({\hat{x}}_{u},{\hat{y}}_{i},\Psi \right)*p\left({\hat{x}}_{u}\right),$$

and Equation (17) can be considered to be a sampling process of the distribution model using probability $\hat{p}\left({\hat{x}}_{u}\right)$. Define

(18) $$\psi ={\left({\hat{x}}_{u}-{\hat{y}}_{i}\right)}^{T}\Psi^{-1}\left({\hat{x}}_{u}-{\hat{y}}_{i}\right)$$

due to Ψ as a positive definite or semi-definite matrix, so that ψ can be described as follows:

(19) $$\psi ={\left({\hat{x}}_{u}-{\hat{y}}_{i}\right)}^{T}{\left(U\Pi U^{T}\right)}^{-1}\left({\hat{x}}_{u}-{\hat{y}}_{i}\right),$$

where $U=\left[\begin{array}{cc} u_{1} & u_{2} \end{array}\right]$ is a linear transformation and denotes the rotation transformation of the target, and $\Pi =\mathrm{diag}\left(\lambda_{t}^{w},\lambda_{t}^{h}\right)$ represents the width and height of the target, respectively. By ignoring the rotation transformation of the target in the image plane in order to simplify computation, Equation (16) can be simplified as a generic ellipse formula,

(20) $$\begin{array}{c} \psi ={\left({\hat{x}}_{u}-{\hat{y}}_{i}\right)}^{T}\Psi^{-1}\left({\hat{x}}_{u}-{\hat{y}}_{i}\right)=\frac{{\left(\hat{x}-{\hat{x}}_{i}\right)}^{2}}{\lambda_{t}^{w}}+\frac{{\left(\hat{y}-{\hat{y}}_{i}\right)}^{2}}{\lambda_{t}^{h}}=\phi_{t}^{s}, \end{array}$$

whose shape is determined by $\lambda_{t}^{w},\lambda_{t}^{h}$, and $\phi_{t}^{s}$, where $\phi_{t}^{s}$ denotes the Mahalanobis distance and is linearly dependent on the scale of the target.

When estimating the target size, it is necessary to traverse the search window to sample points

(21) $$\lambda_{t}^{w}=\sqrt{\frac{\Sigma_{j=1}^{n}{\left({\hat{x}}_{j}-{\hat{x}}_{i}\right)}^{2}}{n-1}},$$

(22) $$\lambda_{t}^{h}=\sqrt{\frac{\Sigma_{j=1}^{n}{\left({\hat{y}}_{j}-{\hat{y}}_{i}\right)}^{2}}{n-1}},$$

where $\left({\hat{x}}_{j},{\hat{y}}_{j}\right)$ denotes the sample point, and *n* is the number of sample points. The computational complexity is linearly related to the search window size. The tracking speed is significantly reduced in the case of large-scale targets. When the salient CN feature is applied, the back-projection is a sparse distribution. Therefore, a down-sampling strategy is reasonably adopted to sparsely sample the candidate pixels and use the sampling area to estimate the distribution in the entire search window. We perform down-sampling once to detect the target points sparsely in the search window. The sample interval can be selected according to the following empirical formula:

(23) $$v=\max\left(\left[\frac{\min(w,h)}{20}\right],2\right),$$

and we can obtain the equal probability ellipse (only the major axis and the minor axis; the inclination angle is ignored). Using the definition of integral and the elliptic area formula we obtain

(24) $$\begin{array}{c} \phi_{s}^{t}\pi \sqrt{\lambda_{t}^{w}\lambda_{t}^{h}}\approx \int \int \delta \left(p\left({\hat{x}}_{u}\right)-\xi \right)dxdy\approx v^{2}\int \int \delta \left(p\left({\hat{x}}_{u}\right)-\xi \right)d\frac{x}{v}d\frac{y}{v}, \end{array}$$

where the ξ is the confidence threshold. Therefore,

(25) $$\phi_{s}^{t}\approx v^{2}\frac{\int \int \delta \left(p\left({\hat{x}}_{u}\right)-\xi \right)d\frac{x}{v}d\frac{y}{v}}{\pi \sqrt{\lambda_{t}^{w}\lambda_{t}^{h}}}.$$

However, the application of salient features to back-projection inevitably leads to various probability distribution maps. That is, $\lambda_{t}^{w},\lambda_{t}^{h}$ do not represent the actual size of the target, so the estimated width and height should be scaled to the actual size $s_{t}=\left(w_{t},h_{t}\right)$. We introduce the assumption of local and global consistency and the assumption that the target scale does not change drastically, so the size of the target at the *t*-th frame $s_{t}=\left(w_{t},h_{t}\right)$ is obtained as follows:

(26) $$\left\{\begin{array}{c} w_{t}=\frac{\lambda_{t}^{w}\sqrt{\phi_{s}^{t}}}{\lambda_{t-1}^{w}\sqrt{\phi_{t-1}^{s}}}*w_{t-1}\\ h_{t}=\frac{\lambda_{t}^{h}\sqrt{\phi_{s}^{t}}}{\lambda_{t-1}^{h}\sqrt{\phi_{t-1}^{s}}}*h_{t-1}. \end{array}\right.$$

To improve the stability of the scale estimation, we choose linear interpolation to update the target scale by

(27) $$s_{t}^{l}=\left(1-\mu \right)s_{t-1}^{l}+\mu s_{t}.$$

### 4.3. Tracking by Fusion

The estimated position and scale of the target can be computed by using the above two methods. However, the target position obtained by the standard kernel-based algorithm will be interfered by objects with similar color features in the background so as to result in a position shift. Changes in the target shape and the boundary effect will also cause a shift in the DCF method. Therefore, this paper adopts a particle fusion algorithm based on the Monte Carlo method to improve the accuracy of the estimated position and scale of the target.

The target appearance model in the search window can be expressed in the following form:

(28) $$H^{w}\left(x\right)=\left[\begin{array}{cccc} h_{1}^{w}\left(x\right), & h_{2}^{w}\left(x\right), & \cdots , & h_{n}^{w}\left(x\right) \end{array}\right].$$

We obtain two estimated position candidates via the proposed algorithm and KCF algorithm. The target position in the new frame can be expressed as a weighted sum of these two positions:

(29) $$\bar{P}=\sum_{k=1}^{n}w_{k}P_{k}.$$

We use the Bhattacharyya distance between the appearance model in the search window and the target appearance model as the weights:

(30) $$\rho =-\ln\left(\sum_{i=1}^{n}\sqrt{\left.h_{i}^{w}\left(x\right)h_{i}^{t}\left(x\right)\right.}\right).$$

By assuming that the interference caused by the background conforms to a Gaussian distribution, the weight can be thus computed as follows:

(31) $$w_{k}=\frac{e^{\frac{\left(1-\rho_{k}\right)}{2\sigma^{2}}}}{\sum_{j=1}^{n}e^{\frac{\left(1-\rho_{j}\right)}{2\sigma^{2}}}}.$$

By updating the model at the target position in the new frame, we utilize the same online model updating strategy as other DCF-based trackers by:

(32) $$h^{t}=\left(1-\eta \right)h^{t-1}+\eta h.$$

## 5. Experiments

To evaluate the performance of the proposed tracker, we implemented the proposed method with an Intel i5 2.50 GHz CPU, 16 GB RAM, and a Windows 10 × 64 operating system. The extensive experiments were conducted on the Temple-Color-128 (TC128) [29] dataset. TC128 is a dataset of 128-color video sequences that are used for visual tracking benchmarks. The dataset was created to study the role of color information in visual tracking. The dataset contains various challenging factors, such as occlusion, illumination change, deformation, motion blur, etc. The dataset also provides ground truth bounding boxes and attribute annotations for each sequence. We chose this dataset because it is a comprehensive and representative dataset for evaluating the performance of color-based trackers.

The performance of the algorithms was evaluated by: (i) average overlap ratio (AUC) of the success plot quantifying the result; (ii) the precision rate (PRE), which is the percentage of frames where the center distance between the predicted bounding box and the ground truth bounding box is within a certain threshold (we set it to 20 pixels); and (iii) the frames per second (FPS), which directly reflects the real-time performance of the algorithm. When evaluating the FPS of the algorithm, in order to simulate the real-time tracking scenario, we count the time for reading the image as the processing time.

### 5.1. Implementation Details

Our method was evaluated on TC128 by following the benchmark evaluation protocol [9]. All parameters of the algorithm were fixed for all video sequences.

For the PSACF, μ and η in Formulas (27) and (32) were set to 0.2 and 0.02, respectively. The confidence threshold ξ in Equation (24) was set to 0.25. Other parameters not specified were set the same as those for KCF.

### 5.2. Experiments and Results

#### 5.2.1. Ablation Study

To verify the effectiveness of each component of the proposed algorithm, we conducted ablation studies with different modules enabled. Specifically, these modules include: (i) a scale estimation module (SE) based on a classic appearance model and probability estimation, where the color name feature is adopted as the color feature; (ii) down-sampling module (DS), where the sample interval is obtained by (23); and (iii) salient color feature filter module (SC), which uses the filtered color feature histogram instead of the classic background weighted histogram model.

We conducted experiments on Temple-Color-128. The overall evaluation result is presented in Table 1. The baseline method without any components (NONE) has the lowest PRE and AUC. However, it also has the highest frame rate of 59.2853 FPS. The method with SE has a slightly higher PRE and AUC than the baseline method. The SE can improve the performance of tracking, but at the cost of computational efficiency. The results of the method with SE + DS show that DS can reduce the computational cost and increase the tracking speed, especially for large-scale targets, but at a cost of some accuracy loss. The method with all components enabled has the highest precision rate and average overlap ratio among all the methods. It also has a slightly lower frame rate than that of the method with scale estimation and down-sampling only. This shows that salient color name filters can effectively improve the performance, but at a small cost to computational efficiency. Overall, our method with all components SE + DS + SC has a superior performance in terms of accuracy and robustness compared to the other methods. This demonstrates that all components play an important role in the method.

#### 5.2.2. Evaluation of Sequences

**Figure 2:**
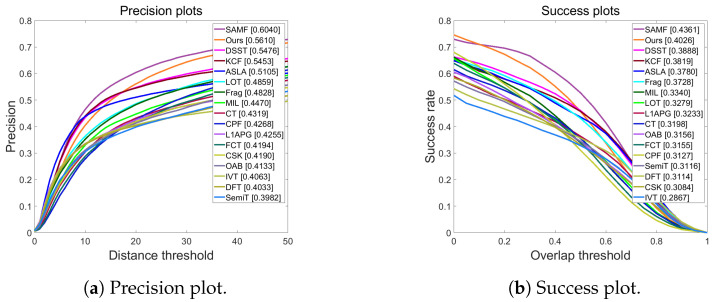
Evaluation of the TC128 dataset.

TC128 data are used for comparison among the proposed PSACF and other popular algorithms, as illustrated in Table 2. Figure 2 shows the success plot and precision plot. The proposed method achieves the second-best performance in terms of the AUC and PRE metric, which means that our method is more accurate and robust than the others. It also shows that our method can effectively handle various challenging factors, such as occlusion, illumination change, deformation, etc.

The proposed method also achieves a high frame rate of 44.9217 FPS, which is much faster than most of the other methods, except for KCF and DFT. This indicates that our method is more efficient and practical.

The proposed method has a slightly lower precision rate than SAMF. The DS module may introduce some errors in estimating the target position. However, this difference is not very significant, and the proposed method still has a high precision rate of 0.5610. This shows that our method can still provide reliable and consistent position estimates for the target.

Overall, the PSACF has a superior performance in terms of accuracy, robustness, efficiency, and speed compared to the other methods. This demonstrates the effectiveness and innovation of the proposed algorithm.

#### 5.2.3. Performance Analysis

**Figure 3:**
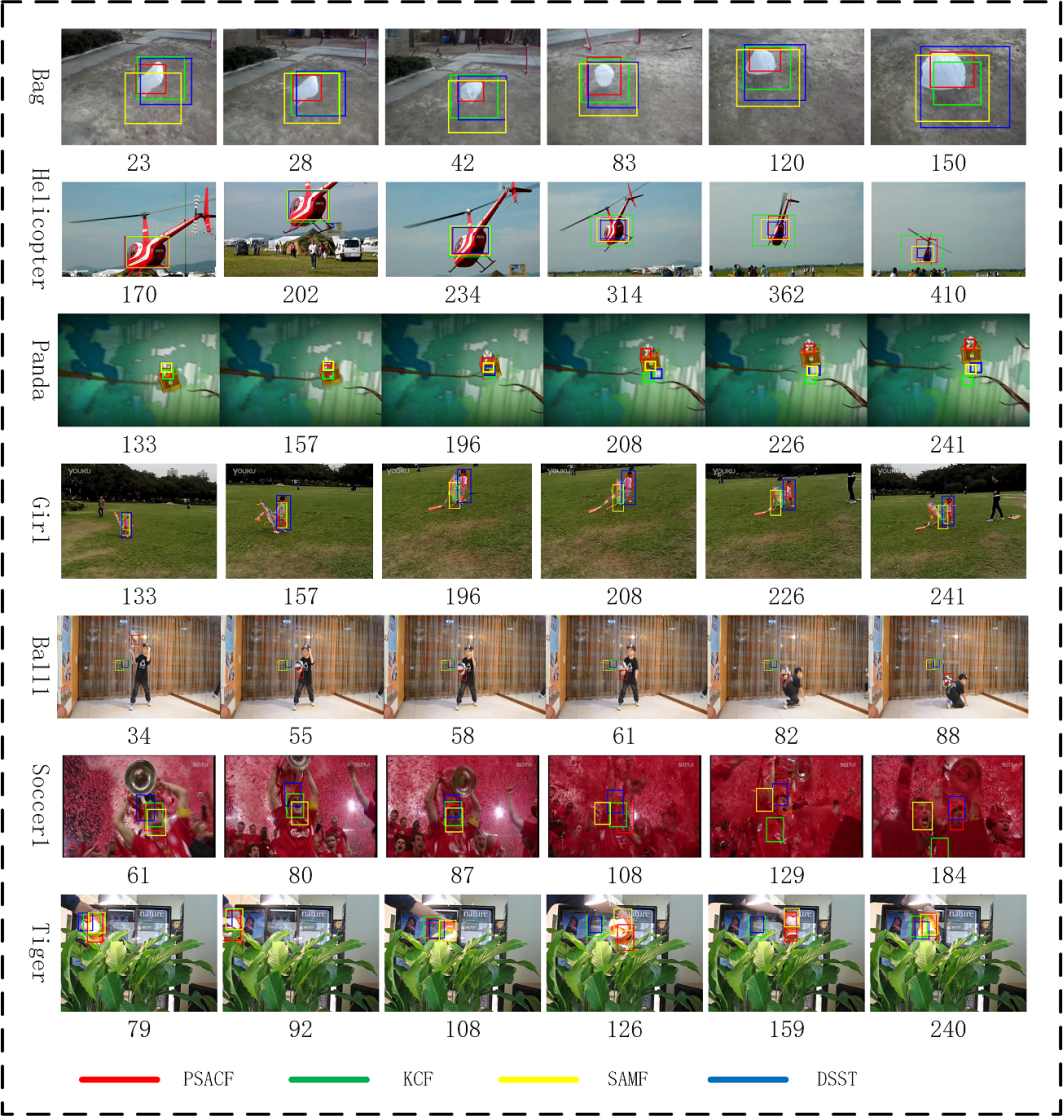
Illustration of the qualitative tracking results on challenging sequences.

In Figure 3, some representative samples are given to analyze the performance of the proposed algorithm.

From the sequences Bag and Helicopter, it can be observed that our method can not only handle the scale variation but also adapt to the shape change of the target. When the target is non-rigidly deformed, the aspect ratio of the bounding box will vary accordingly. However, other algorithms are not sensitive to the nonrigid deformation of the target.

From the sequences Panda and Ball, it can be seen that the proposed algorithm can track fast-moving targets and is robust to motion blur and fast deformation. At the same time, the proposed algorithm will also reduce the influence of boundary effects.

From the sequences Tiger and Soccer1, the PSACF algorithm has superior long-term tracking abilities compared to other methods. Our scale estimation method can also locate the center of the salient features of the target, and the center is used to update the state. When the correlation filter tracker is affected by the boundary effect and deviates from the tracking target, we select the optimal results for integration according to the long-term target template maintained in memory.

**Figure 4:**
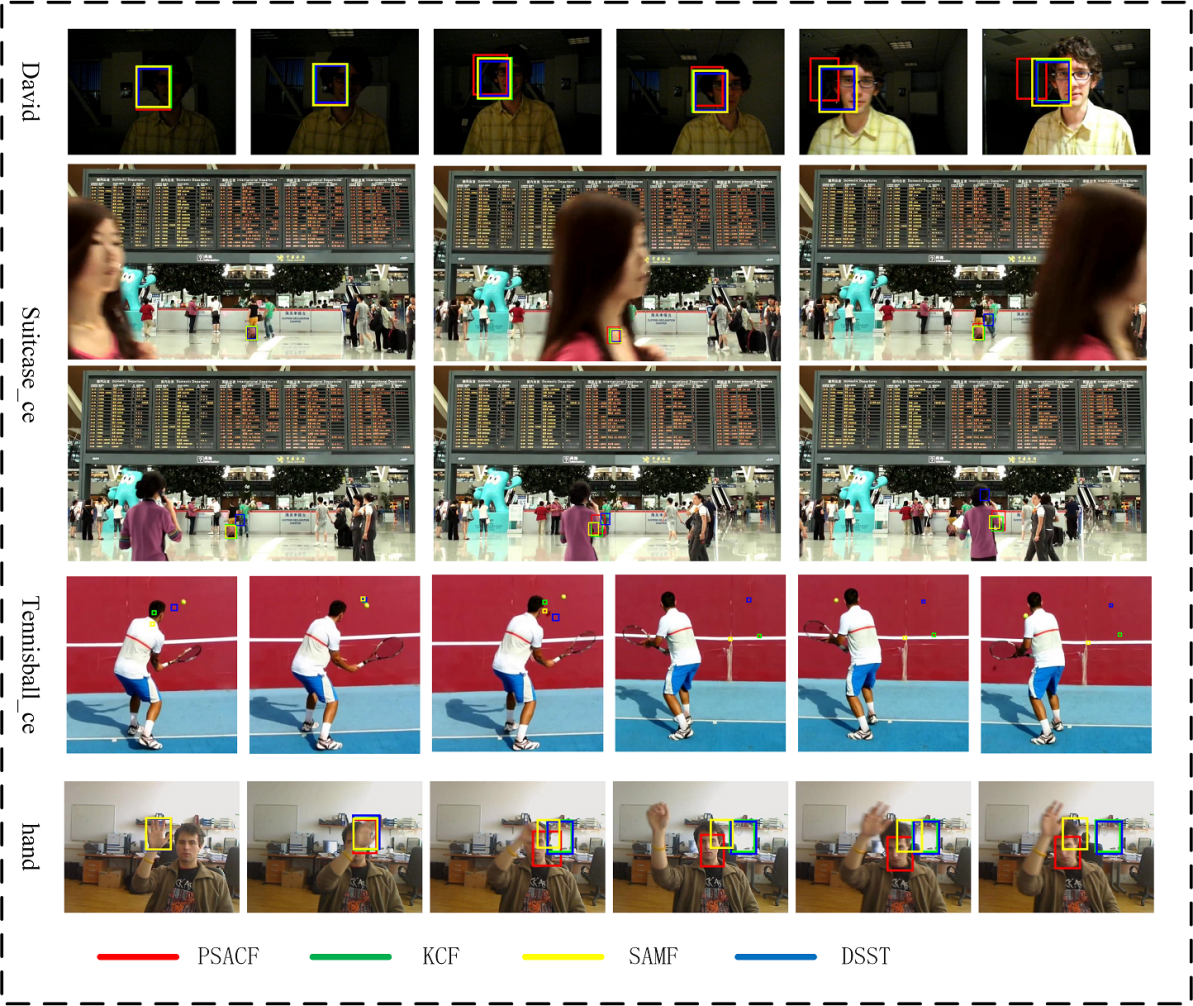
Some failure cases on challenging sequences.

Figure 4 shows some failure cases of the PSACF tracker. In the first row, the illumination changes drastically, which causes our appearance model to lose track of the target. The fusion tracking technique reduces the boundary effect if the illumination is stable. Additionally, if the scale of the target changes rapidly, especially when the illumination undergoes significant variation, our method will not be able to precisely estimate the size of the target. However, the SAMF, KCF, and DSST trackers can still track the target because they exploit the texture feature, which is insensitive to illumination change, for scale estimation. In the second and third rows, the target underwent complete occlusion for a few frames and several seconds, respectively. Every tracker could handle short-term occlusion but cannot deal with long-term complete occlusion, which also resulted in tracking failure. In the fourth row, no tracker could handle tiny targets. In the last row, the target underwent non-rigid deformation in a short time span, which resulted in tracking failure.

## 6. Conclusions

In this paper, we addressed the problem of scale-adaptive tracking in computing power-constrained applications. We used color name (CN) features and a salient feature to reduce the target appearance model’s dimensionality. We then estimated the target scale based on a Gaussian distribution model and introduced global and local scale consistency assumptions to restore the target’s scale. We fused the tracking results with the DCF-based tracker to obtain the new position and scale of the target. Our research contributes a novel and efficient scale-adaptive tracking method that can be applied to various computing-constrained scenarios, such as embedded systems, edge computing, or mobile devices. However, our method still has some limitations and challenges that need to be addressed in future work. For example, our method may fail to track objects with long-term complete occlusion or in scenarios with drastic illumination changes. We plan to explore more robust latent information in target appearance methods, as well as more adaptive fusion strategies, to improve our method’s performance in these challenging situations.

## Figures and Tables

**Table 1:** Ablation study of the proposed method. SE, DS, and SC represent scale estimation, down-sampling, and salient color name filters, respectively.

Components	PRE	AUC	FPS
NONE	0.5310	0.3388	59.2853
SE	0.5574	0.3415	40.3942
SE + DS	0.5408	0.3568	46.5514
SE + DS + SC	0.5610	0.4026	44.9217

**Table 2:** Comparison experiment of the proposed method.

Methods	AUC	PRE	FPS
PSACF(Ours)	0.4026	0.5610	44.9217
SAMF	0.4361	0.6040	12.9108
DSST	0.3888	0.5476	15.5754
KCF	0.3819	0.5453	59.2853
CPT	0.3127	0.4268	4.1844
CT	0.3198	0.4319	8.9621
DFT	0.3114	0.4033	58.7570
FCT	0.3155	0.4194	35.2754
FragTrack	0.3728	0.4828	19.4681
IVT	0.2867	0.4063	8.3748
L1APG	0.3233	0.4255	2.9720
MIL	0.3340	0.4470	4.5498
OAB	0.3156	0.4133	37.5747

## Data Availability

The data that support the findings of this study are available from the corresponding author, upon reasonable request.
